# Supplementary material for: Evidence against tetrapod-wide digit identities and for a limited frame shift in bird wings
Source: Nat Commun. 2019 Jul 19;10:3244. doi: 10.1038/s41467-019-11215-8 (PMC6642197; doi:10.1038/s41467-019-11215-8)
Supplement: Supplementary file 1 — Supplementary Information [file 41467_2019_11215_MOESM1_ESM.pdf]

Supplementary Information

Evidence against tetrapod-wide digit identities and for a limited frame shift in bird wings

Stewart et al.

Table of contents

Supplementary Figure 1 | Analyses of full transcriptomes do not show clustering of samples by digit.

Supplementary Figure 2 | Analyses of all transcription factors show patterns of sample clustering consistent with analyses of limb patterning genes.

Supplementary Figure 3 | Clustering analyses of chicken hindlimb digit transcriptomes.

Supplementary Figure 4 | Differential expression analyses of adjacent chicken hindlimb digits.

Supplementary Figure 5 | Comparing fold change of differentially expressed genes to random genes.

Supplementary Figure 6 | Correspondence of chicken forelimb and hindlimb digits.

Supplementary Figure 7 | Evaluating how differential expression analyses are impacted by variance of replicates.

Supplementary Figure 8 | Mean Dispersion values of the digit transcriptomes.

Supplementary Figure 9 | Summary of the data sampled.

Supplementary Figure 10 | Differential expression analyses of adjacent digits without PC1 correction of *Anolis* data.

Supplementary Table 1 | Human and mouse phenotypes have been described for 28 of the 49 CDEGs.

Supplementary References

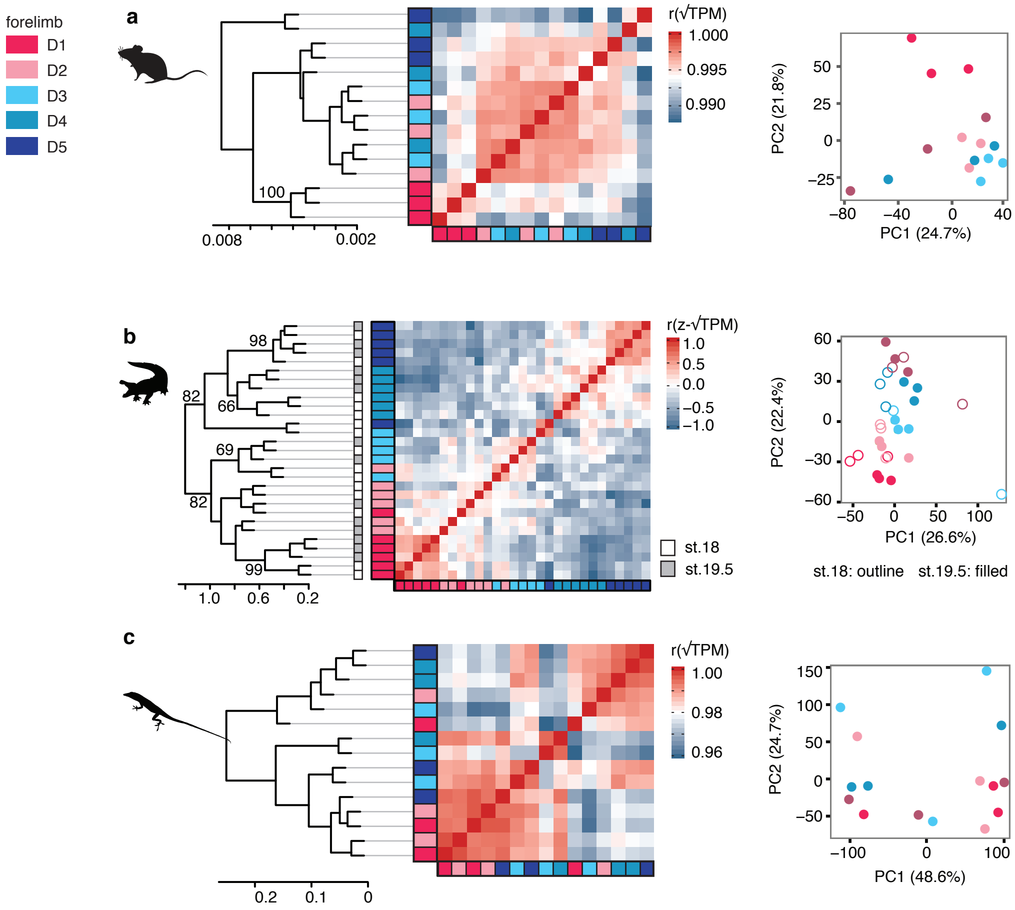

Supplementary Figure 1 | Analyses of full transcriptomes do not show clustering of samples by digit. (a) In mouse, only D1 forms a cluster in HCA, indicating that similarity can be diagnosed when the expression of all genes is considered; more-posterior digits do not form clusters of replicates. (b) In alligator, a cluster with low bootstrap support is observed for D4 samples. Stage 18 and stage 19.5 samples are differentiated as filled or outlined points in PCA plot. (c) In *Anolis,* samples do not reveal stable clustering of digit replicates. Alligator illustration reproduced with permission by Michael Richardson. *Anolis* illustration by Sarah Werning (license [https://creativecommons.org/licenses/by/3.0/]) without modification.

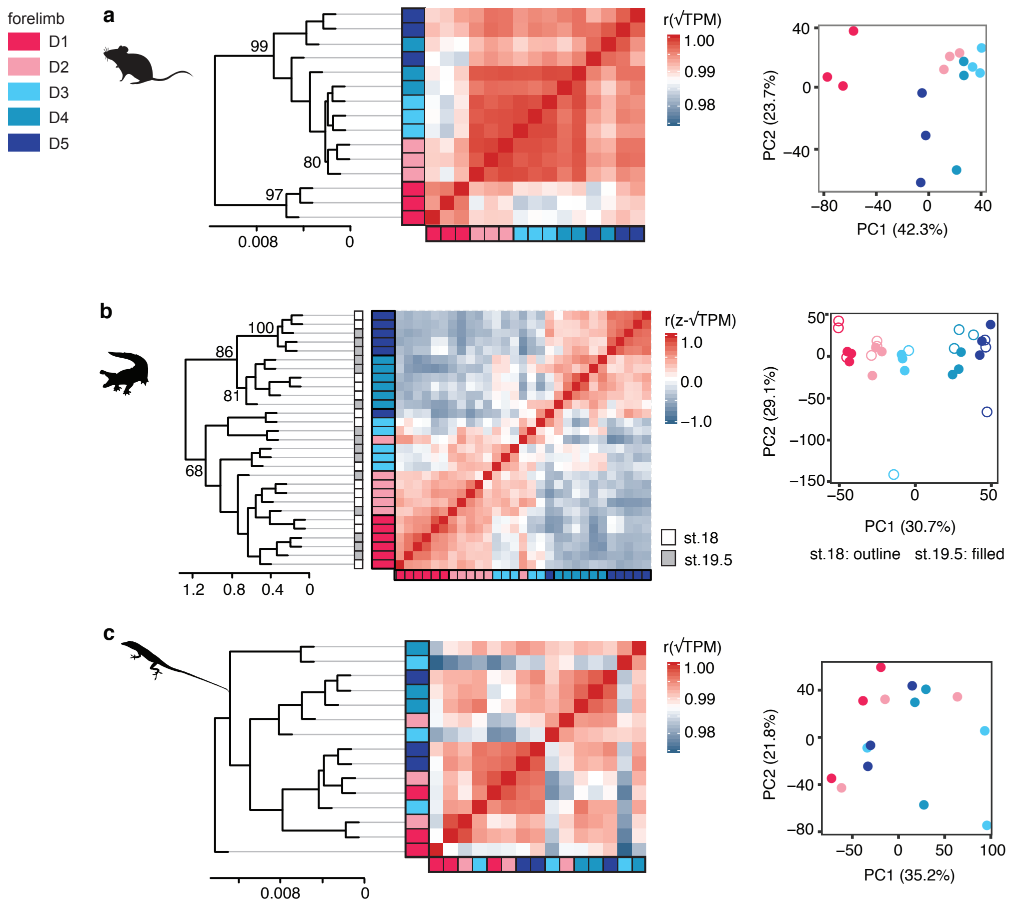

Supplementary Figure 2 | Analyses of all transcription factors show patterns of sample clustering consistent with analyses of limb patterning genes. (a) In mouse, D1 is markedly distinct from the posterior digits. (b) In alligator, two major clusters of digits are observed: (D1, D2, D3)(D4, D5). Stage 18 and stage 19.5 samples are differentiated as filled or outlined points in PCA plot. (c) In *Anolis*, digits do not show gene expression differentiation when all transcription factors are considered. Broadly, adjusted uncertainty values recovered by HCA are lower for a given cluster (*e.g.*, alligator D4) than when limb patterning genes are analyzed. Alligator illustration reproduced with permission by Michael Richardson. *Anolis* illustration by Sarah Werning (license [https://creativecommons.org/licenses/by/3.0/]) without modification.

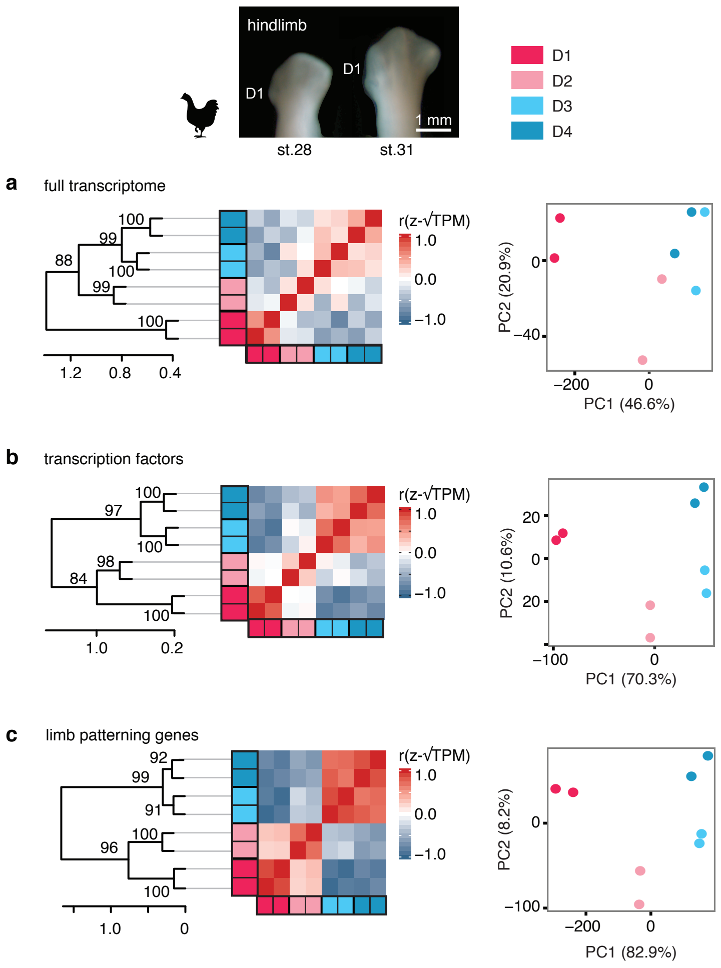

Supplementary Figure 3 | Clustering analyses of chicken hindlimb digit transcriptomes. PCA, heatmap of Pearson's correlations and HCA of (a) the full transcriptome and (b) transcription factor genes, (c) and known limb patterning genes. Each digit is represented by two data points, which correspond to one sample from stage 28 and another from stage 31. Clustering analyses show stable gene expression profiles for individual digits over this developmental window, even after phalangeal number has been established. Chicken illustration reproduced with permission by Michael Richardson.

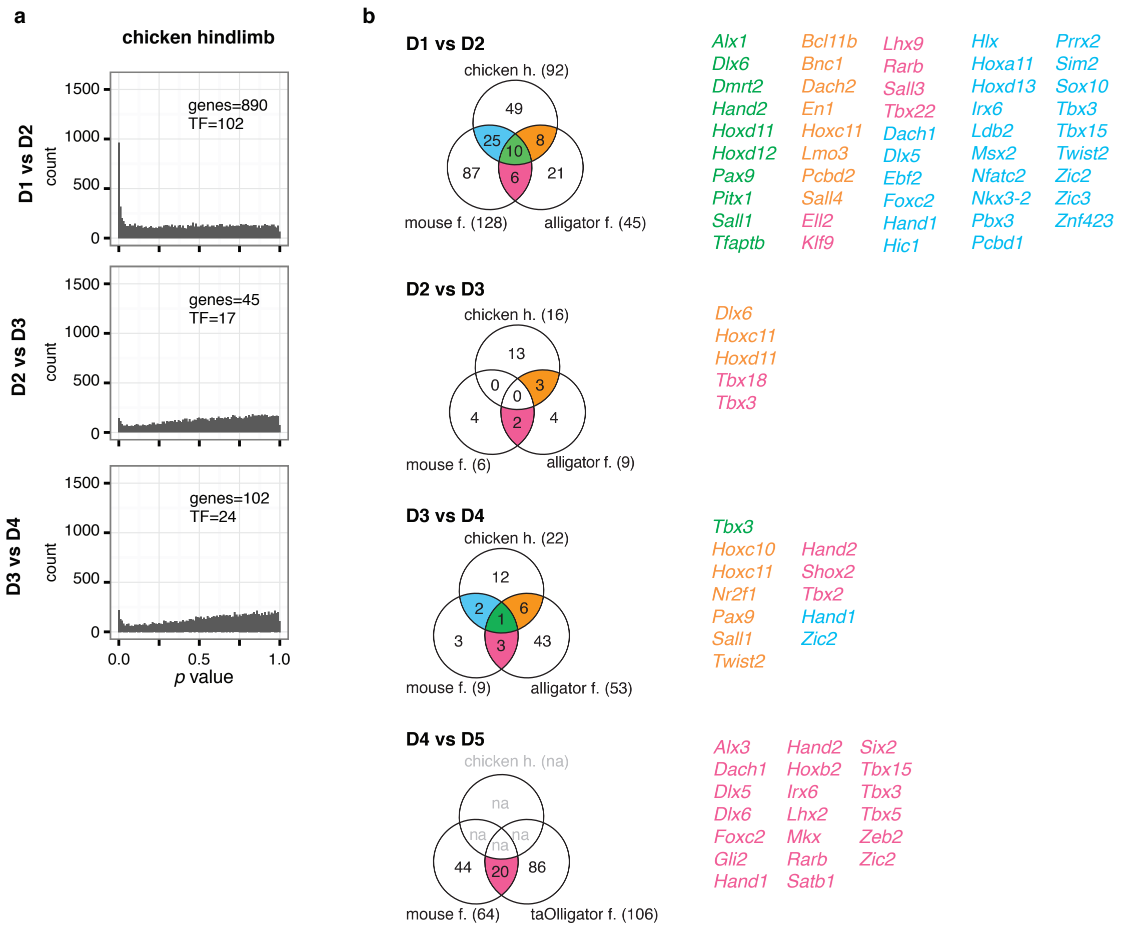

Supplementary Figure 4 | Differential expression analyses of adjacent chicken hindlimb digits. (a) *p* value distributions for pairwise tests of differential expression. The number of genes that are identified as differentially expressed at a FDR threshold of 0.05 are noted in each panel as “genes” and of these the number of transcription factors are noted in each panel as “TF.” (b) Venn diagrams showing the genes that are one-to-one orthologous transcription factors and differentially expressed in each species to a FDR threshold of 0.05.

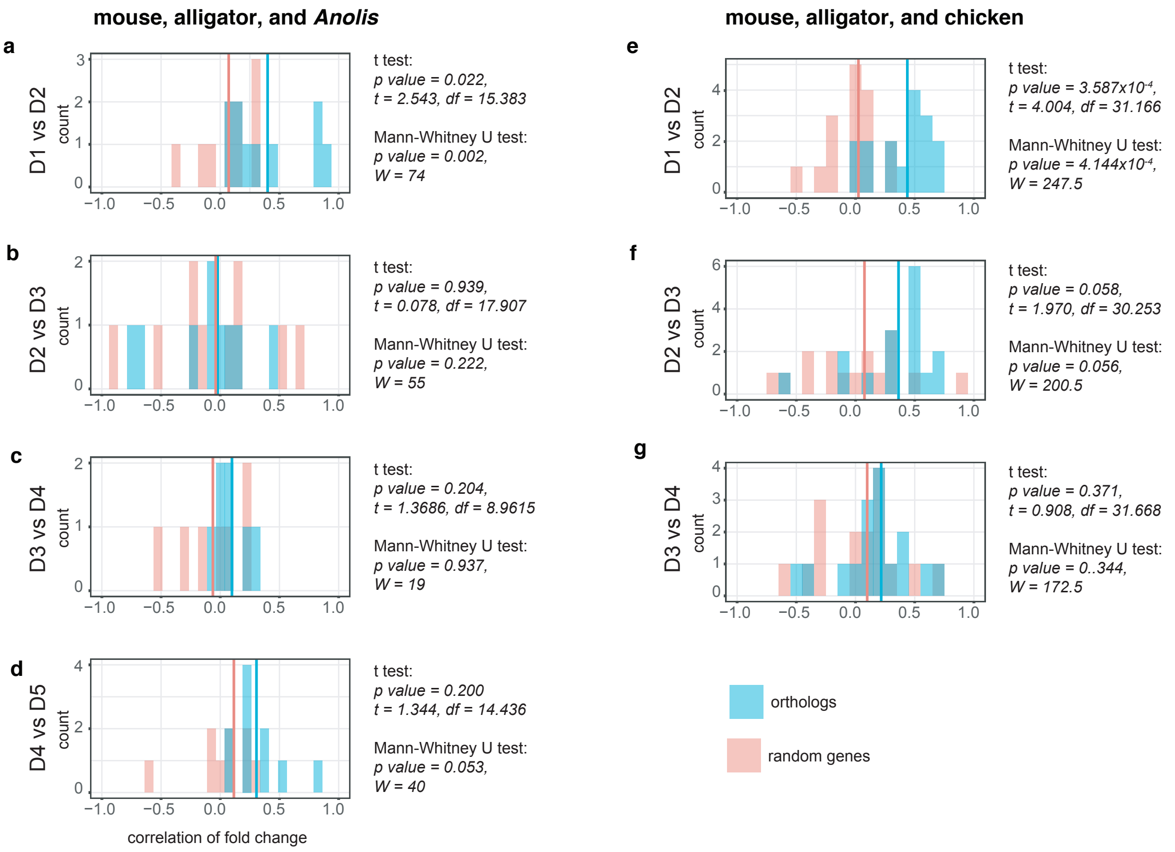

Supplementary Figure 5 | Comparing fold change of differentially expressed genes to random genes. Fold change of genes differentially expressed between adjacent digits for one species was compared to those of orthologous genes in other species and also to randomly selected genes of similar expression level for the other species. Comparisons were made among the pentadactyl limbs (a-d), and also considering chicken, rather than *Anolis* (e-g). Broadly, genes differentially expressed between D1 and D2 behave consistently between species. Among more posterior digits there is limited evidence for conserved behavior. The genes number of genes recovered as differentially expressed at each position for each species are reported in Fig 3. and Supplementary Figure 4 b. Vertical lines in each plot represent the mean values of correlation among comparisons between the sets of orthologous or random genes.

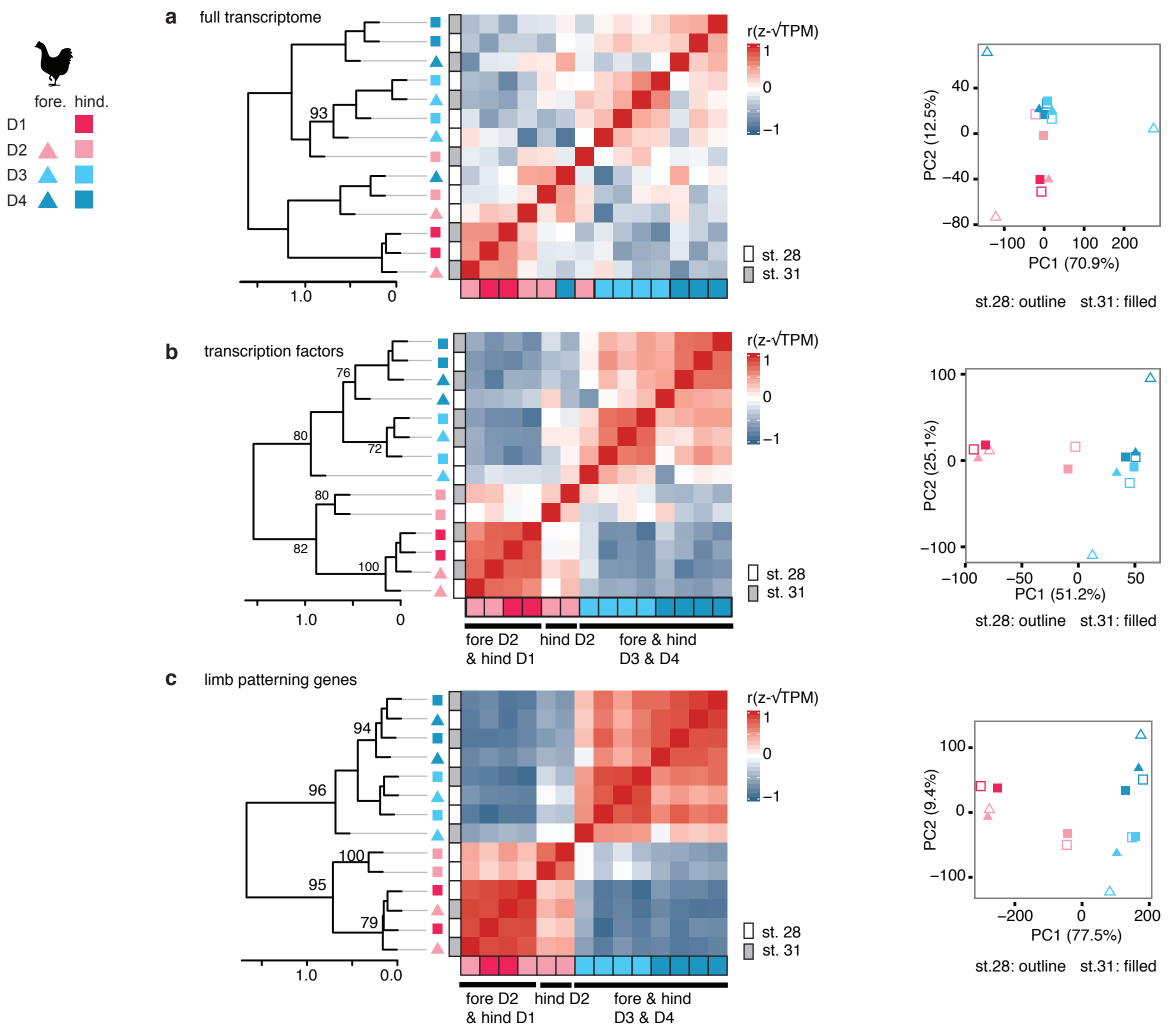

Supplementary Figure 6 | Correspondence of chicken forelimb and hindlimb digits. (a) PCA and HCA of full transcriptomes does not reveal correspondence between digits of the fore- and hindlimb. However, analyses of (b) all transcription factors and (c) limb patterning genes show that the three digits in the avian wing correspond to hindlimb digits D1, D3, and D4. Stage 28 and 31 samples are differentiated as filled or outlined points in PCA plot. . Chicken illustration reproduced with permission by Michael Richardson.

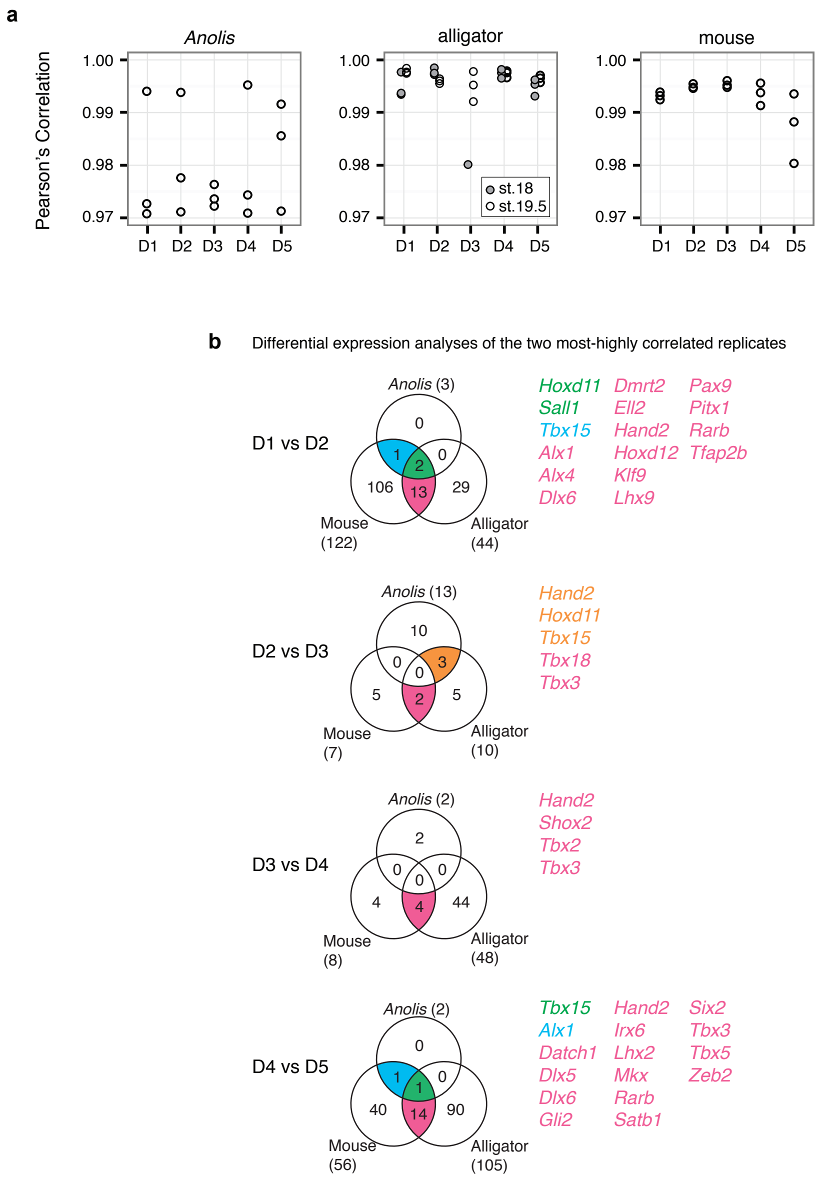

Supplementary Figure 7 | Evaluating how differential expression analyses are impacted by variance of replicates. (a) Pearson's correlation values of sample replicates showing that in *Anolis* replicates are less highly correlated than the replicates of mouse and alligator; however, for the digits D1, D2, D4 and D5 two of the three replicates are correlated with values comparable to the other species (>0.99). Therefore, to assess whether variance in *Anolis* was biasing our analyses, tests of differential expression were replicated using only the two most-highly correlated replicates of each digit. (b) Venn diagrams of one-to-one orthologous transcription factors genes identified as differentially expressed between adjacent digits with a FDR threshold of 0.05 when differential expression analyses considered only the two most-highly correlated samples of each digit.

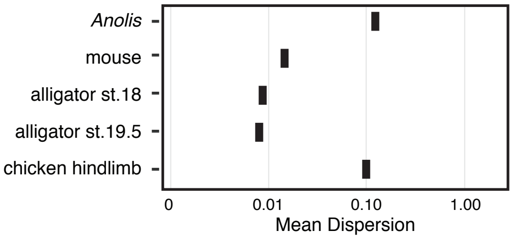

Supplementary Figure 8 | Mean dispersion values of the digit transcriptomes. Values calculated by edgeR showing that although pedigree likely impacts tests of differential gene expression, it does not explain the homogeneity of the *Anolis* digits.

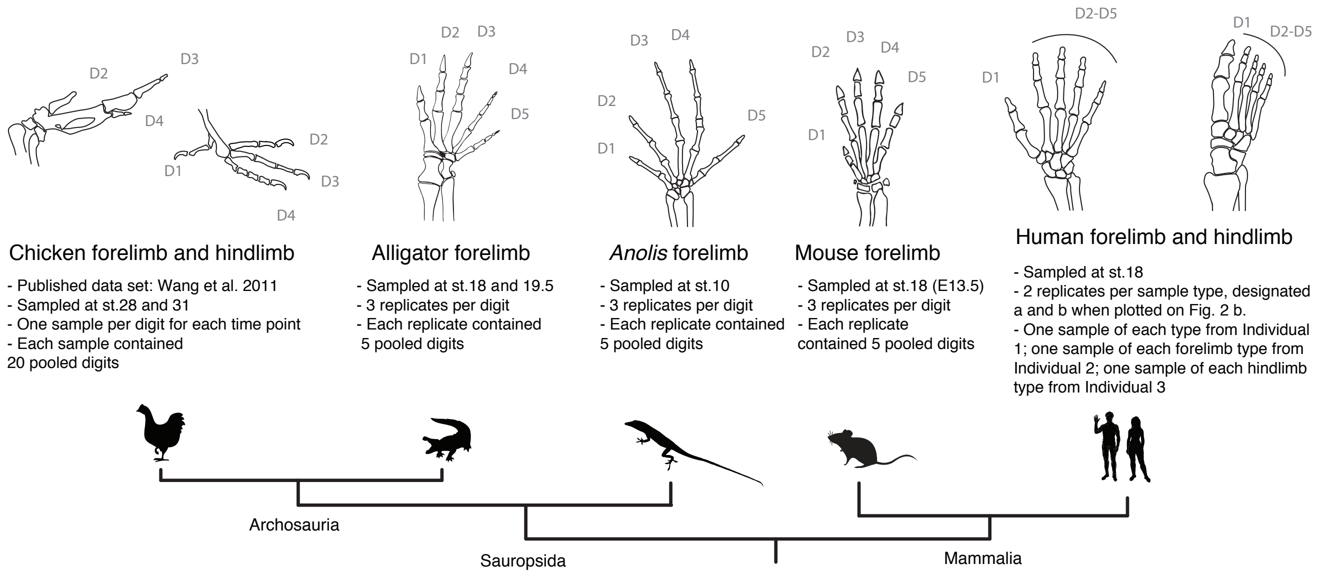

Supplementary Figure 9 | Summary of the data sampled. Illustrations are of the adult skeletons from the dorsal perspective, anterior is left. 'Replicates' refer to biological replicates. Illustration of mouse forelimb skeleton[1] was replicated and modified from journal Development with permission. Illustrations of the alligator forelimb skeleton, and chicken and alligator silhouettes[3] were replicated with permission by Michael Richardson. *Anolis* illustration by Sarah Werning without modification (license: https://creativecommons.org/licenses/by/3.0/).

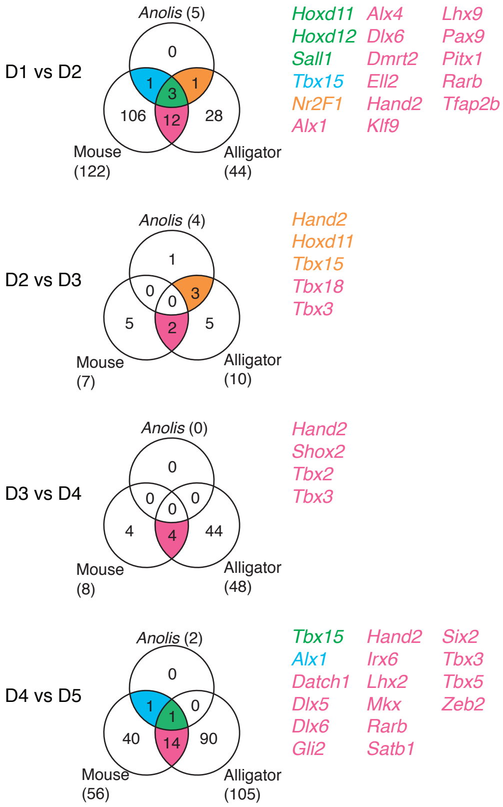

Supplementary Figure 10 | Differential expression analyses of adjacent digits without PC1 correction of *Anolis* data. Venn diagrams of one-to-one orthologous transcription factors genes for mouse, alligator, and *Anolis* that were identified as differentially expressed between adjacent digits with a FDR threshold of 0.05.

Supplementary Table 1 | Human and mouse phenotypes have been described for 28 of the 49 CDEGs

gene	human phenptype or syndrome	mouse phenotypes
Alx1	Camptodactyly[5]	polydatyly[7]
Dlx5	Split hand/foot malformation[4,6]	ectrodactyly, monodactyly, syndactyly[7]
Dlx6		brachydactyly, ectrodactyly, syndactyly[7]
En1		25 phenotypes including adactyly, ectopic digits, polydactyly, syndactyly, truncation of digits, fused phalanges[7]
Ets2	Chitayat syndrome[6]	
Hand1		hypoplastic limb buds[7]
Hand2		19 phenotypes including oligodactyly, polydactyly, abnormal pollex morphology[7]
Hic1	Hand-foot-genital syndrome[4]	abnormal fore- and hindlimb morphology[7]
Hoxa13	Guttmacher syndrome[4,5], Hand-foot - genital syndrome[4,6], Postaxial hand polydactyly[5]	21 phenotypes including brachydactyly, clinodactyly, syndactyly[7]
Hoxd11		7 phenotypes including abnormal phalanx, abnormal and fused carpals[7]
Hoxd12		16 phenotypes including brachyphalangia, brachydactyly, clinodactyly, oligodactyly[7]
Hoxd13	Brachydactyly-syndactyly syndrome[4], VACTERL association, Brachydactyly type E[5]	29 phenotypes including brachydactyly, ect rodactyly, polydactyly, polysyndactyly[7]
Nkx3-2	Spondylo-megaepiphyseal-metaphyseal dysplasia[6]	
Pax9		polydactyly, polysyndactyly[7]
Pitx1	Clubfoot and Lower limb malformations[4], Liebenberg syndrome[6]	11 phenotypes including brachydactyly, oligodactyly, and clubfoot[7]
Runx2	Brachydactyly[4], MDMHB[6], cleidocranial dysplasia (which includes abnormal thumbs and brachydactyly)[7]	10 phenotypes including abnormal phalanx morphology[7]
Sall1	Townes-Brocks Syndrome[4,5,6], Lenz microphthalmia syndrome[5]	10 phenotypes including oligodactyly, preaxial polydactyly, syndactyly and triphalangia[7]
Satb2	chromosome 2q32-q33 deletion syndrome (which includes clinodactyly archnodactyly, and Talpes equinovarus[7]	
Sox10	kallmann-syndrome[6], Klein-Waardenburg syndrome[6], PCWH syndrome (which includes Pes cavus)[7]	
Tbx2	17q23.1q23.2 microdeletion syndrome[5]	Polydactyly, postaxial polydactyly[7]
Tbx3	Ulnar-mammary syndrome[4], Limb-mammary syndrome[5], post-axial polydactyly[5]	16 phenotypes including oligodactyly[7]

Tbx5	Holt-Oram syndrome[4,5]	10 phenotypes including abnormal phalanx morphology[7]
Tbx15	Cousin syndrome (which includes brachydactyly)[4,5]	9 phenotypes including abnormal phalanx morphology[7]
Tfap2b	Char syndrome[4,5]	Polydactyly, postaxial polydactyly[7]
Trps1	Trichorhinophalangeal syndrome type 1[4,5], type 2[5], and type 3[5]	
Twist2	Ablepharon macrostomia syndrome[5]	
Zic2		6 phenotypes mostly restricted to carpals[7]
Zic3	VACTERL association[5], Radial abnormalities[5], aplasia/Hypoplasia of the radius[5]	

Supplementary References

1 Krebs, O. et al. Replicated anterior zeugopod (*raz*): a polydactylous mouse mutant with lowered *Shh* signalling in the limb bud. *Development* **130**, 6037-6047, doi: 10.1242/dev.00861 (2003).

2 Muller, G. B. & Alberch, P. Ontogeny of the Limb Skeleton in *Alligator mississipiensis*: Developmental lnvariance and Change in the Evolution of Archosaur Limbs. *J. Morph.* **203**, 151-164 (1990).

3 de Bakker, M. A. G. et al. Digit loss in archosaur evolution and the interplay between selection and constraints. *Nature* **500**, 445-448 (2013).

4 Stenson, P.D. et al. The Human Gene Mutation Database (HGMD®): 2003 Update. *Hum. Mutat*. **21**, 577-581 (2003).

5 NIH Genetic and Rare Disease Information Center. http://rarediseases.info.nih.gov.

6 National Library of Medicine (US). Genetics Home Reference [Internet]. https://ghr.nlm.nih.gov (2013).

7 Blake J.A. et al. Mouse Genome Database (MGD)---2017: community knowledge resource for the laboratory mouse. *Nucl. Acids Res.* **4,** D723-D729 (2017).
