# Supplementary material for: Evidence against tetrapod-wide digit identities and for a limited frame shift in bird wings
Source: Nat Commun. 2019 Jul 19;10:3244. doi: 10.1038/s41467-019-11215-8 (PMC6642197; doi:10.1038/s41467-019-11215-8)
Supplement: Supplementary file 3 — Reporting Summary [file 41467_2019_11215_MOESM3_ESM.pdf]

nature research

Corresponding author(s): Thomas Stewart and Gunter Wagner

Last updated by author(s): Jun 30, 2019

Reporting Summary

Nature Research wishes to improve the reproducibility of the work that we publish. This form provides structure for consistency and transparency in reporting. For further information on Nature Research policies, see Authors & Referees and the Editorial Policy Checklist .

Statistics

For all statistical analyses, confirm that the following items are present in the figure legend, table legend, main text, or Methods section.

n/a	Confirmed	
☐	☒	The exact sample size (*n*) for each experimental group/condition, given as a discrete number and unit of measurement
☐	☒	A statement on whether measurements were taken from distinct samples or whether the same sample was measured repeatedly
☐	☒	The statistical test(s) used AND whether they are one- or two-sided<br>*Only common tests should be described solely by name; describe more complex techniques in the Methods section.*
☐	☒	A description of all covariates tested
☐	☒	A description of any assumptions or corrections, such as tests of normality and adjustment for multiple comparisons
☐	☒	A full description of the statistical parameters including central tendency (e.g. means) or other basic estimates (e.g. regression coefficient) AND variation (e.g. standard deviation) or associated estimates of uncertainty (e.g. confidence intervals)
☐	☒	For null hypothesis testing, the test statistic (e.g. *F*, *t*, *r*) with confidence intervals, effect sizes, degrees of freedom and *P* value noted<br>*Give P values as exact values whenever suitable.*
☒	☐	For Bayesian analysis, information on the choice of priors and Markov chain Monte Carlo settings
☒	☐	For hierarchical and complex designs, identification of the appropriate level for tests and full reporting of outcomes
☐	☒	Estimates of effect sizes (e.g. Cohen's *d*, Pearson's *r*), indicating how they were calculated

Our web collection on statistics for biologists contains articles on many of the points above.

Software and code

Policy information about availability of computer code

Data collection	No software was used to collect data.
Data analysis	The following packages were used in this study and implemented with Python (v2.7.2): Tophat2 (v2.0.6), HTSeq (v0.5.3p). The following packages were used in this study and implemented with R R (v3.3.1): EdgeR (Release 3.1), All code used for analyses is available on GitHub [https://github.com/ThomasAStewart/digit_identity_project].

For manuscripts utilizing custom algorithms or software that are central to the research but not yet described in published literature, software must be made available to editors/reviewers. We strongly encourage code deposition in a community repository (e.g. GitHub). See the Nature Research guidelines for submitting code & software for further information.

Data

Policy information about availability of data

All manuscripts must include a data availability statement. This statement should provide the following information, where applicable:

- Accession codes, unique identifiers, or web links for publicly available datasets
- A list of figures that have associated raw data
- A description of any restrictions on data availability

The RNA-sequencing data for mouse, alligator and Anolis (including counts of mapped reads), is available on Gene Expression Omnibus (GEO) repository under accession number GSE108337 [https://www.ncbi.nlm.nih.gov/geo/query/acc.cgi?acc=GSE108337]. Sequencing data for human limb samples is available through the database of Genotypes and Phenotypes (dbGaP) under study accession number phs001226.v1.p1 [https://www.ncbi.nlm.nih.gov/projects/gap/cgi-bin/study.cgi?study_id=phs001226.v1.p1]. Supplementary data files include: Supplementary Data 1, unique gene IDs corresponding to all gene lists described; Supplementary Data 2, median gene lengths; Supplementary Data 3, bootstrap values for all PCA plots; Supplementary Data 4, mapped reads for chicken. The source data underlying Figs 4b,c and 6c are provided as a Source Data file.

Field-specific reporting

Please select the one below that is the best fit for your research. If you are not sure, read the appropriate sections before making your selection.

- [x] Life sciences
- [ ] Behavioural & social sciences
- [ ] Ecological, evolutionary & environmental sciences

For a reference copy of the document with all sections, see nature.com/documents/nr-reporting-summary-flat.pdf

Life sciences study design

All studies must disclose on these points even when the disclosure is negative.

Sample size	Sample sizes for RNA-seq were selected according according to references 29 and 30, which recommend at least three biological replicates for differential expression testing,
Data exclusions	RNA extraction of two samples of alligator stage 18 digit D3 yielded too little RNA for our sequencing approach. Therefore, these samples were not included in the study.
Replication	A previous paper by our group (Wang et al. 2011, Nature doi:10.1038/nature10391) assessed replication of the RNA-sequencing experimental approach used here. Specifically, two of the samples in that study were resampled and resequenced. In each case, the correlation coefficient of the samples was above 0.99.
Randomization	Experimental groups were comprised of specimens from the same species. Statistical analyses for differential expression studies focused only on comparisons of samples within each group (i.e., comparing differential expression of the digit of a species to another digit of that same species), therefore covariates from these groups are not relevant to the study.
Blinding	Investigators were not blinded to the group allocation during the experiment or when assessing outcomes. It was not possible to blind experimenters during tissue collection (dissection of embryos of different species). The same statistical analyses were applied to samples of each species, and therefore blinding is not relevant to data analysis.

Reporting for specific materials, systems and methods

We require information from authors about some types of materials, experimental systems and methods used in many studies. Here, indicate whether each material, system or method listed is relevant to your study. If you are not sure if a list item applies to your research, read the appropriate section before selecting a response.

Materials & experimental systems

n/a	Involved in the study	
x		Antibodies
x		Eukaryotic cell lines
x		Palaeontology
	x	Animals and other organisms
x		Human research participants
x		Clinical data

Methods

n/a	Involved in the study	
x		ChIP-seq
x		Flow cytometry
x		MRI-based neuroimaging

Animals and other organisms

Policy information about studies involving animals; ARRIVE guidelines recommended for reporting animal research

Laboratory animals	Mice embryos were collected from a pregnant female of the strain C57BL/6J (Jackson Laboratories). Anolis embryos were collected from a breeding colony at Loyola University.
Wild animals	*Provide details on animals observed in or captured in the field; report species, sex and age where possible. Describe how animals were caught and transported and what happened to captive animals after the study (if killed, explain why and describe method; if released, say where and when) OR state that the study did not involve wild animals.*
Field-collected samples	*For laboratory work with field-collected samples, describe all relevant parameters such as housing, maintenance, temperature, photoperiod and end-of-experiment protocol OR state that the study did not involve samples collected from the field.*
Ethics oversight	Protocols for mouse care and euthanasia were approved by Yale University's Institutional Animal Care and Use Committee (protocol #2015-11-483). Protocols for Anolis care and breeding were approved by Loyola University's Institutional Animal Care and Use Committee (protocol #1992). All methods were performed in accordance with relevant local guidelines and regulations.

Note that full information on the approval of the study protocol must also be provided in the manuscript.
