# Supplementary material for: Evidence against tetrapod-wide digit identities and for a limited frame shift in bird wings
Source: Nat Commun. 2019 Jul 19;10:3244. doi: 10.1038/s41467-019-11215-8 (PMC6642197; doi:10.1038/s41467-019-11215-8)
Supplement: Supplementary file 4 — Description of Additional Supplementary Files [file 41467_2019_11215_MOESM4_ESM.pdf]

**Description of Additional Supplementary Files**

File Name: Supplementary Data 1
Description: Unique gene IDs corresponding to all genes in the study.

File Name: Supplementary Data 2
Description: Median transcript lengths for all genes in the study.

File Name: Supplementary Data 3
Description: Bootstrap values for all PCA plots

File Name: Supplementary Data 4
Description: Mapped reads for Chicken RNA-seq data
